# Enhancing treatment outcomes of Alcohol Use Disorder patients through ICT-based cognitive training tools: a randomized controlled trial

**DOI:** 10.1016/j.ijchp.2025.100623

**Published:** 2025-09-23

**Authors:** Rita Costa, Sérgio Lima, Mónica S․ Cameirão, Sergi Bermúdez i Badia, Ana Lúcia Faria

**Affiliations:** a Faculdade de Ciências Exatas e da Engenharia, Universidade da Madeira, Funchal, Portugal; b NOVA Laboratory for Computer Science and Informatics, Lisboa, Portugal; c Agência Regional para o Desenvolvimento da Investigação, Tecnologia e Inovação, Funchal, Portugal; d Casa de Saúde São João de Deus, Instituto São João de Deus, Funchal, Portugal; e Faculdade de Artes e Humanidades, Universidade da Madeira, Funchal, Portugal

**Keywords:** Alcohol use disorder, Cognitive training, Interactive technologies, Minnesota Model for Change

## Abstract

Individuals with alcohol use disorders (AUD) often face cognitive impairments that hinder long-term abstinence. While structured AUD interventions are effective, they don't guarantee abstinence maintenance, and cognitive training (CT) is rarely integrated. CT could be crucial for rehabilitating these deficits and preventing relapse, especially if personalized.

This randomized controlled trial assessed the impact of adding personalized CT to standard AUD treatment. Sixty participants were randomized into three groups: 1) Virtual Reality (VR) CT using activity-of-daily-living simulations (Reh@City - RC), 2) paper-and-pencil CT via a web platform (Task Generator - TG), and 3) time-matched treatment as usual (TAU). The intervention included 12, 30-minute sessions, with pre-, post-intervention, and follow-up neuropsychological assessments.

At post-intervention, experimental groups showed broader cognitive improvements. The RC group improved in naming, executive functions, fluency, inhibitory control, processing speed, attention, and visual memory. The TG group showed gains in general cognitive functioning, executive functions, abstraction, sensitivity to interference, processing speed, attention, and visual memory. The TAU group's improvements were observed in executive functions, abstraction, processing speed, attention, and visual memory.

Personalized cognitive training is effective for AUD-related cognitive deficits. The VR approach (RC) particularly impacted inhibitory control, processing speed, and sustained attention—domains critically affected in this population. Future research should explore the CT role in preventing relapse.

## Introduction

Alcohol use disorder (AUD) is a chronic condition that impacts multiple domains of life, including social, familiar, and occupational functioning, with a significant negative effect on the individual’s health. According to the National Health Inquiry (SICAD, 2021), about half of the Portuguese population reported daily (21 %) or frequent (37 %) alcohol consumption in 12 months. Regarding binge consumption (consuming six or more units of alcohol on a certain occasion), 43 % of alcohol consumers reported at least one episode of binge drinking in the past year, and part of this sample reported weekly (11 %) or daily (3 %) episodes of binge drinking. In 2021, almost 30,000 individuals were hospitalized due to problems related to alcohol consumption (of direct or secondary cause) (SICAD, 2021), representing a significant burden to the healthcare system. The existing literature indicates that about 50 % to 80 % of formally diagnosed AUD individuals demonstrate cognitive and behavioral alterations when compared with healthy peers; 30 % to 40 % of them with enough deficits to fulfil clinical impairment diagnosis (Nixon, 2020).

Furthermore, approximately 10 % of abstinent individuals with AUD have a diagnosis of an organic brain disorder such as Korsakoff syndrome (KS) or alcohol-induced dementia (Bates et al., 2013; Nixon, 2020).

A recent study exploring the course of cognitive performance during the inpatient treatment of 524 patients revealed that more than half (284) of this population presented mild neurocognitive impairment, and 169 had major cognitive impairments with diagnosis criteria for Korsakoff`s syndrome (Bruijnen et al., 2021). According to the literature, these impairments are found in different cognitive domains and are prevalent not only during short-term abstinence but also in long-term abstinence (Stavro et al., 2013; Zahr et al., 2011; Zou, 2018). Although there is evidence that sustained abstinence is linked to improvements in neurocognitive functioning (Bernardin et al., 2014; Mulhauser et al., 2018), a significant percentage of AUD patients have long-term cognitive impairments that can compromise the treatment outcomes (Bates et al., 2013; Bruijnen et al., 2021; Miller, 1991). A meta-analysis exploring cognition in AUD patients and consequential impairments indicates that a heterogeneous pattern of deficits is prevalent. Still, AUD seems to mostly affect specific domains related to memory and executive functioning, such as attention, inhibitory control, decision-making (Oscar-Berman et al., 2014), verbal fluency, processing speed, working memory, problem-solving, inhibition/impulsivity, verbal learning, verbal memory, visual learning, visual memory, and visuospatial abilities (Stavro et al., 2013). These deficits have been described in a significant amount of research and seem to be linked to brain structural and functional changes in the prefrontal and temporal brain regions, which have been observed in individuals with prolonged and abusive alcohol consumption (Zahr et al., 2011). Importantly, the prevalent impairments in specific prefrontal cortex functions, such as response inhibition and salience attribution (a syndrome that causes excessive salience to drug-related cues), can be one explanation for the chronically relapsing disorder and maintenance of the cycle of addiction (Goldstein & Volkow, 2011). A systematic review explored the potential impact of general cognition and executive functioning on treatment outcomes for different substance abuse (Domínguez-Salas et al., 2016). This review’s main conclusions highlight that deficits in general cognitive and executive functioning (decision-making, inhibitory control) are associated with poorer addiction treatment outcomes, meaning poor adherence to treatment and higher rates of relapse (Domínguez-Salas et al., 2016).

In Portugal, the standardized treatment approach is based on a multidisciplinary model and well-established international guidelines: the Minnesota Model for Change (Cook, 1988). In this approach, individuals with AUD are submitted to a structured intervention program that contributes to organic detox while training psychological and social skills. Cognitive training (CT) is not typically part of the standard intervention with AUD, which is not coherent with the outcomes from the studies referenced above and may partially explain the fact that a significant percentage of AUD patients present chronic relapses and are not able to maintain long-term abstinence.

The fact that cognitive deficits are not properly addressed in AUD interventions can be somewhat explained by the insidious onset of alcohol-related deficits in this population; these impairments are frequently overlooked in both clinical and nonclinical settings (Bates et al., 2013). Other important factors have to be addressed, namely the lack of a sufficient number of experts to perform such a resource-intensive intervention. In the last decades, Information and Communication Technologies (ICTs) have been demonstrated to be a promising solution to this problem, facilitating access to this type of approach (Restrepo et al., 2020). Off-the-shelf ICTs are becoming more accessible in terms of price and availability, and their validation with clinical populations has grown exponentially over the years (Faria et al., 2016; Mazza et al., 2021; Restrepo et al., 2020). Specifically in addictive behaviors, different studies have used ICTs, such as Virtual Reality (VR), to implement Cue Expose therapy (VR enables a more ecological context) (Mazza et al., 2021), but also for CT protocols. For instance, a recent study from Gamito and colleagues explored the feasibility of a CT approach using activities-of-daily-living VR-based tasks to improve AUD patients’ cognitive functioning while undergoing treatment in a therapeutic community (Gamito et al., 2021). According to the authors, the VR-based CT promoted significant improvements in attention and mental flexibility at post-intervention. A study with AUD patients exploring the impact of a working memory (WM) training paradigm on cognitive functions revealed that the training group improved in verbal WM. This result did not affect other neuropsychological tasks and did not significantly affect the individuals with heavy drinking patterns (Khemiri et al., 2019a). Other recent reviews about CT mediated by ICTs for AUD concluded that ICTs CT and rehabilitation can improve not only the adherence and motivation for treatment but also can have a positive impact on self-esteem, self-efficacy and daily functioning. Although with promising evidence, these results need consolidation and more consistency across studies and intervention protocols (Restrepo et al., 2020).

Given the growing accessibility and advantages of ICTs, they should be considered a promising tool to address AUD patients' cognitive impairments and stimulation needs. Some of the important benefits of technology in this specific area are 1) the possibility to develop tools that have ecological validity, 2) providing safe and controlled training environments, 3) the activities can be personalized, and 3) providing immediate feedback (Faria et al., 2016). Some of these VR scenarios that replicate activities of daily living, such as shopping or action planning, reveal superior validity and reliability in cognitive assessment and rehabilitation (Bourgeois et al., 2023; Faria et al., 2023). A randomized controlled trial found that stroke patients using a VR-based intervention (Reh@City) showed greater improvements in global cognitive functioning, attention, and executive functions when compared to conventional therapy (Faria et al., 2016).

A review exploring the role of cognitive training in the treatment of AUD revealed that CT protocols can enhance performance and generalize gains to similar tasks. However, transfer effect and functional outcomes are usually not reported in the existing literature (Nixon & Lewis, 2019).

Although individuals with AUD often show improvements with CT, the degree to which this training improves diverse aspects of cognitive performance and functional outcomes, such as drinking and psychosocial adaptation, is unclear. The high heterogeneity in terms of sample characteristics, training protocols and outcome measures makes it difficult to generalize and compare the real effect of CT in AUD treatment (Nixon & Lewis, 2019).

Considering the importance of including cognitive training in AUD standard treatment, in this study, we aim to explore the efficacy and specific benefits of two content equivalent CT tools: VR simulations of activities of daily living [Reh@City (RC)] and paper-and-pencil tasks generated from a web platform [Task Generator (TG)] in comparison to treatment as usual (TAU). These CT tools, initially developed and validated for stroke patients (Faria et al., 2020), originated a cognitive rehabilitation platform with challenges designed to address multidomain cognitive functions often impaired after stroke. The tasks mimic activities of daily living, such as shopping at a supermarket, pharmacy, or clothing store; performing instrumental activities of daily living at home like cooking, cleaning, or moving around a city (for a more detailed review, see Faria et al., 2016, 2020). These tools can be used with other clinical populations with cognitive impairments, and they have already been clinically validated with patients with mental health and behavioral disorders (Câmara et al., 2023). In previous studies, these tools have been shown to be effective, and the VR approach revealed a superior impact in important aspects, namely a better transference of the cognitive improvements to their health, well-being and quality of life. In this study, we aim to explore how could each of these approaches (paper and pencil and VR) enhance the treatment of AUD individuals and impact their health, well-being and quality of life. For this, we have defined three main research questions (RQ):

**RQ 1: Does the inclusion of cognitive training in the treatment of AUD have an impact on the improvement of memory, executive functions, and attention outcome measures?**
**RQ 2: Does ecologically valid cognitive training have more clinical impact in terms of cognition, health, well-being and quality of life than traditional paper-and-pencil training?**
**RQ 3: Does cognitive training as a complementary intervention have an impact on abstinence maintenance?**

## Materials and methods

### Sample recruitment

**Fig. 1:**
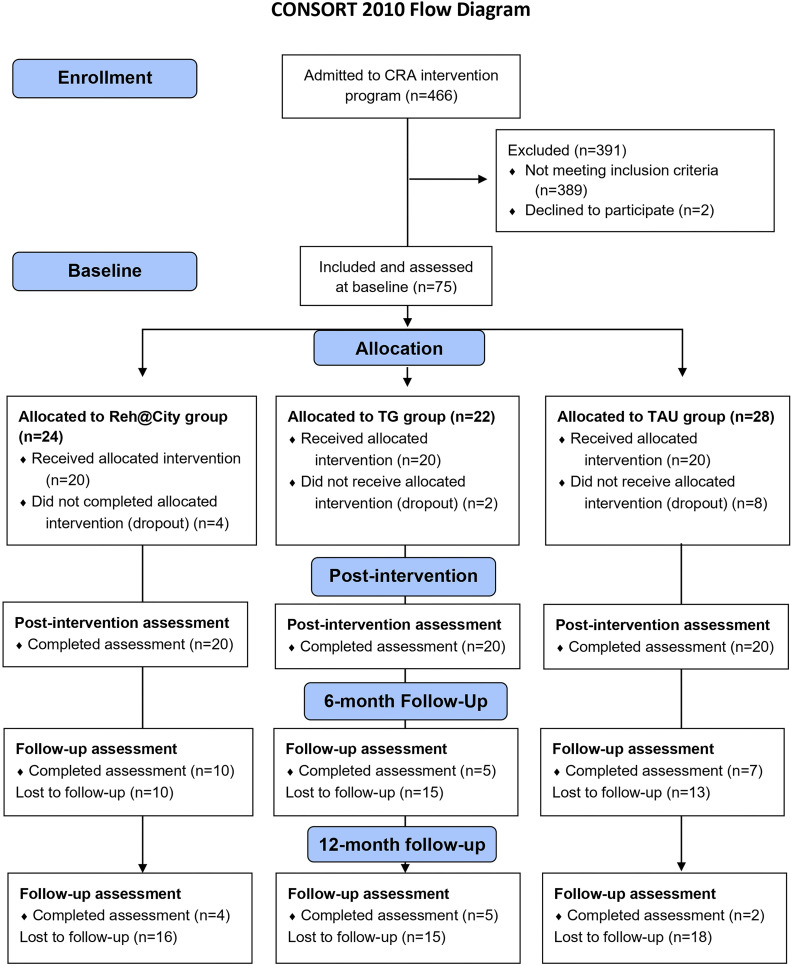
CONSORT flow diagram of the RCT.

The sample recruitment occurred from October 2018 to August 2020 at Casa de Saúde São João de Deus (CSSJD), Ricardo Pampuri Recovery Center for AUD, in Madeira Island, Portugal. All the participants were inpatients of the four-week treatment, twelve-step program based on the Minnesota Model (Cook, 1988). Following the admission, patients were screened for the study inclusion criteria: (a) have a diagnosis of AUD and be attending the inpatient program; (b) age between 35 and 65 years old [(this age range was intentionally selected to align with the typical demographic profile of individuals undergoing treatment at the rehabilitation center. Furthermore, it allowed us to focus on AUD-related cognitive deficits, which can manifest on early ages, depending on consumption, while minimizing confounding from severe age-related neurocognitive decline (Oscar-Berman et al., 2014; Oscar-Berman & Marinković, 2007); (c) be able to read and write; (d) have normal or corrected-to-normal vision and hearing; standard neuropharmacological therapy and (e) be motivated to participate (assessed via brief clinical interview during screening to ensure voluntary and engaged adherence). Patients with other substance abuse and/or psychiatric or neurological disorders (present or past) were excluded from participation in this study. Fig. 1 presents the CONSORT flow diagram for this trial.

Overall, this trial included a total of 60 participants (54 men, 6 women), randomly allocated to one of the three groups: TG, RC, and TAU (Table 1).

**Table 1:** Demographic and alcohol consumption data description of the three groups (presented as Mean and SD).

	Virtual Reality Group (*n* = 20)	Paper-and-pencil Group (*n* = 20)	Treatment as usual Group (*n* = 20)
Age	49 (SD= 5.2)	49 (SD= 8.1)	49 (SD= 6.8)
Schooling (years)	6.75 (SD= 2.8)	7.15 (SD= 3.5)	7.15 (SD= 3.1)
Year of Dependence	16.35 (SD= 12.5)	18.6 (SD= 14.2)	16.9 (SD= 10.7)
Value of consume (gr/ml)	244.9 (SD= 128)	337.1 (SD= 254.4)	366.5 (SD= 193)
Number of Admissions (CRA)	2.4 (SD= 1.7)	2.5 (SD= 1.8)	1.95 (SD= 1.3)
AUDIT scale	28,6 (SD= 5.7)	31,4 (SD= 5.4)	30,3 (SD= 5.5)

The CONSORT diagram (Fig. 1) illustrates participant flow. Lost to follow-up cases, particularly at the six and 12-month assessments, were due to contextual challenges. These included significant impacts from the COVID-19 pandemic (e.g., restricted in-person visits, altered rehabilitation routines), coupled with the inherent difficulties in maintaining long-term engagement and abstinence within this population.

### Intervention description

#### Task generator: paper-and-pencil cognitive training

The Task Generator is a free web tool that provides a paper-and-pencil cognitive training program personalized to the patient’s cognitive profile, as assessed by a cognitive screening tool. TG automatically generates a set of different tasks after parametrization. The personalization requires cognitive assessment data to define the levels for the other cognitive domains (attention, memory, executive function, language, and difficulty); after this, a set of 11 tasks is generated and can be downloaded and printed (see Fig. 3 panels c and d). The TG tasks: cancellation, numeric sequences, problem-solving, association, comprehension of contexts, image pairs, word search, mazes, categorization, action sequencing, and memory of stories and pictures, were developed after a participatory design process with rehabilitation professionals (Faria & Bermúdez i Badia, 2015; Faria et al., 2018).

#### Reh@City v2.0: VR-based intervention

The VR-based intervention, the Reh@City was developed using the same TG tasks with an adaptation to a virtual city context, including eight scenarios as streets, buildings, parks, and shops (see Fig. 3 panels a and b). A table detailing the correspondence between paper-and-pencil tasks (TG) and VR tasks (Reh@City v2.0), adapted from previous studies involving stroke (Faria, Pinho & Bermúdez i Badia, 2020), and psychiatric patients (Câmara et al., 2023), is available in the supplementary material section (supplementary material A). The cultural adaptation of Reh@City stimuli such as typical Portuguese supermarket products and brands, are essential details to enhance engagement and familiarity, facilitating skills generalization and increasing ecological validity (for a review, see Paulino et al., 2019 & Faria, Pinho & Bermúdez i Badia, 2020). Reh@City allows a familiar environment to perform the cognitive tasks, being a more ecological training experience, where patients can perform different ADLs: buy food in a supermarket, withdraw money at the bank ATM, set a table at home, or read the newspaper at the kiosk. Throughout the performance, participants have information displayed on the desktop, i.e., the task instructions and different direction cues (e.g., arrows or a GPS-like map indicating the destination); these cues disappear according to the difficulty level but can be presented again if needed, pressing specific keyboard buttons. In the Reh@City screen, a point counter is presented, used as a visual feedback element according to the task completion and participants’ progress.

#### Personalization process

Reh@city v2.0 and Task Generator tools can both be personalized using the same criteria. In this study, the Montreal Cognitive Assessment (MoCA) (Nasredinne et al., 2005) was used to provide an overview of cognitive functioning in residents of a therapeutic community (Marceau et al., 2016). After the personalization according to the MoCA results, the parametrization was conducted using the following model: a mean performance score (0–100 %) was determined after a completed training set in order to establish the difficulty level for the next session and the following criteria was used: (a) the difficulty level was decreased by 0.5 if the mean performance was below 50 %; (b) the difficulty was maintained if the mean performance ranged from 50 to 70 % and (c) the difficulty level was increased by 0.5 if the mean performance was higher than 70 % (Faria and Bermúdez i Badia, 2018). In the Reh@City v2.0 this process of difficulty parameters adaptation is automatically performed (Faria et al., 2016).

### Experiment setup

#### Task Generator

Task Generator is a freely available online application available at https://neurorehabilitation.m-iti.org/TaskGenerator/. Through this website, clinicians can personalize the cognitive training task content according to the described procedure. After personalization, a set of 11 cognitive tasks in PDF format is generated, incorporating training tasks for memory, attention, executive functions, and language. To use this tool, a computer with internet access, a PDF reader, and a printer are required. With the paper-and-pencil tasks printed, patients complete them with the assistance of trained psychologists.

#### Reh@City v2.0

**Fig. 2:**
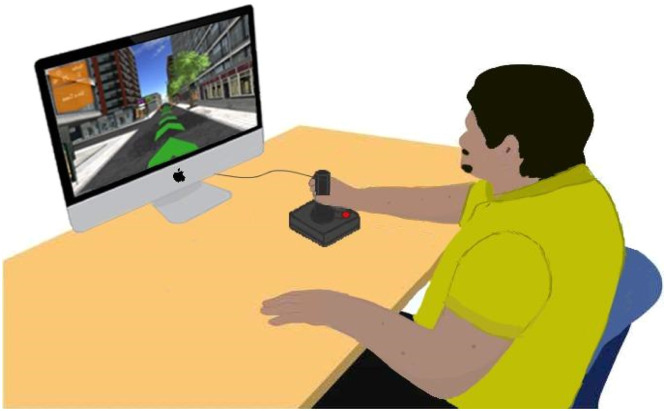
Reh@City v2.0 experimental setup. The user faces an iMac 24 inches monitor and moves the joystick with both hands to interact with the virtual scenario.

The Reh@City v2.0 was implemented using the Unity 3D game engine and installed on an iMac computer with 24 inches. Patients work on a tabletop, facing an LCD monitor, and interact with the virtual environment through a joystick handle (*classic controller)* with two buttons, one for selection and another for moving in different directions (Fig. 2). Considering the available resources and the participant`s potential low digital literacy, a simplified user interface (PC and joystick) was the best option for this trial.

**Fig. 3:**
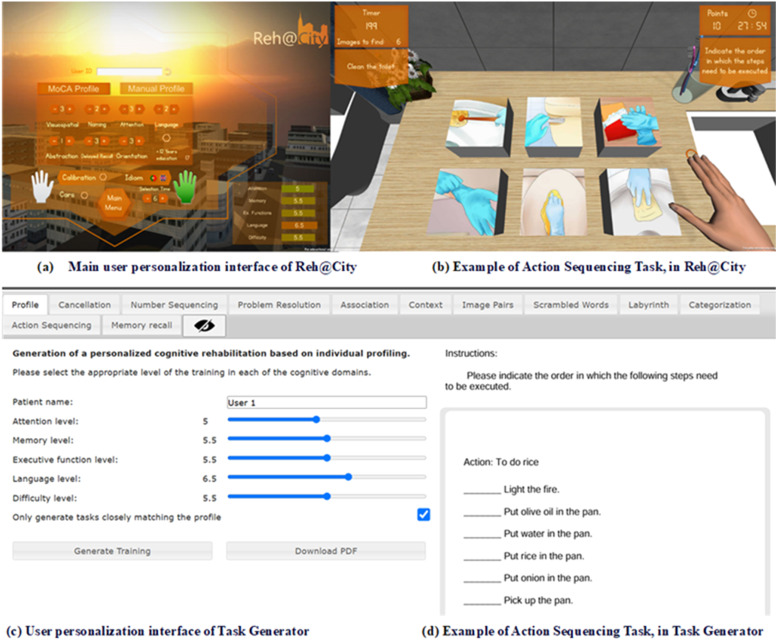
User personalization interface of Reh@City (a), example of Action Sequencing Task, in Reh@City (b) TaskGenerator user personalization interface (c), and an example of Action Sequencing Task, in TaskGenerator (d).

### Trial design

This three-arm Randomized Controlled Trial (RCT) was approved by the board of directors of *Casa de Saúde São João de Deus,* and the protocol was registered at ClinicalTrials.gov (NCT04639895). Fig. 1 illustrates the flow of participants in this study: following the admission, participants who met the criteria and were available to participate read and signed the informed consent document. participants were randomly assigned to one of the three groups: VR-based cognitive training (Reh@City v2.0), content equivalent paper-and-pencil cognitive training (Task Generator), and a passive control group that underwent the standard treatment protocol (TAU), using a ratio of 1:1:1, respecting the order of admission at the center and before the baseline assessment. All patients from the three groups experienced the same treatment program for AUD recovery.

Sociodemographic and specific clinical data, such as consumption value (self-reported alcohol use at admission), number of admissions at the recovery center, and years of consumption, were registered. To assess excessive drinking patterns, the Alcohol Use Disorder Identification Test (AUDIT) was used (WHO,1992; score range: 0–40; scores ≥ 20 suggest probable alcohol dependence), and the 21-item self-report Beck Depression Inventory-II (BDI-II) (Beck et al., 1996) for screening depressive symptoms.

The participants in both experimental groups performed 12 cognitive training sessions of 30 min duration, and the standard treatment during the four-week program, being a time-matched intervention. Participants in the control group received time-matched treatment as usual (TAU) for AUD.

### Outcome measures

To comprehensively assess the cognitive domains targeted by our cognitive training (CT) programs and to obtain a more complete neuropsychological profile of our sample, we selected a battery of measures, witch are described in the following section. With this approach, we aimed to achieve a more precise perception of the clinical impact of our intervention across the different cognitive domains. All participants performed a neuropsychological assessment at baseline, post-intervention, six-month follow-up and 12-month follow-up. All the data collected was exclusively used for the study, and anonymity was ensured by using an ID code for each participant

#### Primary outcome measures: global cognitive functioning, executive functions, sustained attention, processing speed, visual and working memory

To assess global cognitive functioning Montreal Cognitive Assessment (MoCA) was used (Freitas et al. 2011). The Frontal Assessment Battery (FAB) was applied to evaluate six executive functioning constructs: conceptualization, mental flexibility, programming, sensitivity to interference, inhibitory control, and environmental autonomy (Lima, Meireles, Fonseca, Castro & Garrett, 2008). To evaluate sustained and selective attention, a ten-minute cancellation test Toulousse-Piéron (TP), was applied (Toulouse and Piéron, 1986). Additionally, to assess processing speed, we used the Wechsler Adult Intelligence Scale III (WAIS-III) subtests Digit Symbol Coding and Symbol Search (Wechsler, 1997a). Visual memory and working memory were assessed with the Rey-Osterrieth Complex Figure Test (RCFT) (Rey 1998) and Letter-number sequencing (WAIS-III), respectively.

#### Secondary outcome measures: health, well-being and quality of life

The quality of life was evaluated with SF-36 Medical Outcome Study- Short Form Health Survey, portuguese adaptation (Ferreira, 2000). This is a self-report questionnaire that comprises 36 questions assessing eight health domains: Functional Capacity, Physical health, Mental health, General health, Pain, Energy/fatigue, Social functioning, and Emotional well-being.

### Statistical analysis

All statistical analyses were performed using the Statistical Package for the Social Sciences version 28 (SPSS Inc., Chicago, IL, USA). As a criterion for significance an α of 0.050 was adopted for all tests. The normality of the data was assessed with the Shapiro–Wilk test. As some data were not normally distributed, non-parametric tests were used to determine the inter-group and between-group differences. For within-group analyses, the Wilcoxon signed rank tests (W) were performed to examine specific pairwise changes in outcome measures over time. We specifically focused on comparisons from: Baseline versus Post-Intervention and Baseline versus Six-month follow-up within each group. This approach was chosen to maximize statistical power and participant retention, as performing a repeated-measures test requiring data from all three time points (e.g., Friedman test) would have led to substantial data loss due to reduced participant numbers at the follow-up assessment. To control for the family-wise error rate across these two pairwise comparisons from baseline, Bonferroni corrections were applied to the p-values from these Wilcoxon tests (adjusted α=0.05/2 = 0.025).

Between-group comparisons were conducted using the two-tailed Mann-Whitney U test to compare demographic characteristics between the groups at baseline and to compare differences in scores for the primary and secondary outcome measures. Effect sizes (r) were computed as *Z*/√*N* on the pairwise comparisons. The criteria for interpretation of the effect were 0.1 = small, 0.3 = medium, and 0.5 = large.

## Results

All groups were equivalent in neuropsychological outcome measures at baseline and demographic characteristics, aged between 35 and 65 years old (*M* = 49; SD= 6,7), with 4 to 15 years of formal education (*M* = 7; SD= 3,1).

**RQ1: Does the inclusion of cognitive training in the treatment of AUD have an impact on the improvement of memory, executive functions, and attention outcomes?**

### Primary outcome measures

#### Global cognitive functioning - MoCA

Table 2 displays the results from the baseline, post-intervention and six-month follow-up assessment moments. The within-groups analysis (baseline versus post-intervention) revealed that the RC group had significantly improved at post-intervention in the naming domain from MoCA [Baseline: Mdn = 3, IQR= 3–3; Post: Mdn = 3, IQR= 2–3; (*W*_(20)_ = 59,00, *Z* = −2236, *p* = 0025, *r = 0,5*)]. The TG group had statistically significantly better results at post-intervention in the MoCA`s total score [Baseline: Mdn = 20, IQR= 19–22,8; Post: Mdn = 23,5, IQR = 20,3–25; (*W*_(20)_ = 146,50, *Z* = 3339, *p* = 0.001, *r = 0,7*)].

**Table 2:** MoCA, FAB, DSC, SS, L/N Sequencing, TP, ROCFT, and SF-36 scores (Medians and Interquartile Ranges) at Baseline, Post-Intervention, and Six-Month Follow-Up (FU).

	Reh@City v2.0			Task Generator			TAU		
	Baseline (*n* = 20)	Post (*n* = 20)	FU (*n* = 10)	Baseline (*n* = 20)	Post (*n* = 20)	FU (*n* = 5)	Baseline (*n* = 20)	Post (*n* = 20)	FU (*n* = 7)
MoCA Total	21,5 (20- 24,8)	23 (20,3- 24,8)	24 (21,5–27)	20 (19- 22,8)	23,5 (20,3- 25)**	21 (17,5–26,5)	21 (18,3- 22,8)	22 (19,5- 23)	23 (21–24)
Visuo-Executive	4 (3- 5)	4 (3- 5)	4,5 (3,8–5)	4 (3- 4)	4 (3- 4,8)	4 (1,5–5)	4 (3,3- 5)	4 (4- 4,8)	4 (4–5)
Naming	3 (3- 3)	3 (2- 3)**	3 (2- 3)	3 (3- 3)	3 (3- 3)	2 (1,5–3)	3 (2,3- 3)	3 (3- 3)	3 (2–3)
Attention	4 (3- 5)	5 (4- 5,8)	6 (4,8- 6)	4 (3- 5)	4 (3- 6)	5 (4–5,5)	4 (3- 5)	4 (3- 5)	4 (3–5)
Language	2 (1- 2)	2 (1- 2,8)	2 (1–2,3)	1 (1- 1,8)	2 (1- 2)	2 (0,5–2,5)	1 (1- 2)	1 (1- 2)	2 (1–2)
Abstraction	2 (1- 2)	2 (1- 2)	1,5 (1–2)	1 (1- 2)	2 (1- 2)	1 (1–2)	2 (1- 2)	1,5 (1- 2)	1 (1–2)
Memory	2 (2- 3)	2 (1- 3)	2 (2–4)	2 (0,3- 3,8)	3 (1,3- 4)	3 (1–3,5)	2 (1- 3)	3 (1,3- 3,8)	3 (3–4)
Orientation	6 (5- 6)	6 (6- 6)	6 (5,8–6)	6 (5- 6)	6 (5,3- 6)	6 (6–6)	6 (5- 6)	6 (5- 6)	6 (6–6)

FAB Total	15 (14,3- 16,8)	16,5 (15,3- 17)**	17 (15–18)	14 (12,2- 16,8)	16 (14,3- 17)**	17 (14,5–18)	15 (13- 15,8)	16 (14- 17,8)**	15 (13–16)
Conceptualization	3 (2- 3)	3 (2,3- 3)	3 (2,8–3)	2 (1,3- 3)	3 (2- 3)**	3 (2,5–3)	2 (2- 3)	3 (2- 3)**	2 (2–3)
Mental flexibility	2 (1- 3)	3 (2- 3)**	3 (1–3)	2 (1- 2)	2 (1- 2)	2 (1–3)	2 (2- 3)	2 (2- 3)	2 (2–3)
Motor programming	3 (3- 3)	3 (3- 3)	3 (3–3)	3 (2,3- 3)	3 (3- 3)	3 (3–3)	3 (2- 3)	3 (2- 3)	3 (2- 3)
Sensitivity to interference	3 (2,3- 3)	3 (3- 3)	3 (3–3)	2,5 (2- 3)	3 (2,3- 3)**	3 (3–3)	3 (2- 3)	3 (2- 3)	3 (3–3)
Inhibitory control	2 (2- 3)	3 (2- 3)**	3 (2,8–3)	3 (2- 3)	3 (2- 3)	3 (2–3)	2 (2- 3)	2,5 (2- 3)	2 (1–3)
Environmental autonomy	3 (3- 3)	3 (3- 3)	3 (3–3)	3 (3- 3)	3 (3- 3)	3 (3–3)	3 (3- 3)	3 (3- 3)	3 (3–3)

DSC Coding	33 (23,5- 42,3)	39 (27,8–50)**	36,5 (32,5–47)	30,5 (23,3- 42,8)	34,5 (26,3- 48,8)	36 (22,5–45)	33,5 (24- 37,8)	37 (26,3- 45,8)**	48 (20–57)
DSC Incidental Learning	8,5 (4,5–12)	10 (6–15,5)**	9 (5,5–14,5)	6 (4–11,8)	10 (4,5–12)	5 (4–6)	7 (4,5–10,8)	10 (4,5–13,8)	8 (6–16)
DSC Free recall	6 (5–7)	7 (6–8,8)**	7 (6–8)	6 (4,3–7)	6,5 (6–8)**	8 (3–9,5)	7 (5–8)	7,5 (5–8,8)	7 (5–8)

Symbol Search	14 (13- 21,5)	19 (15- 23)**	21 (18–28)	15,5 (9- 20)	14,5 (12- 21,8)**	18 (12,5–19,5)	16,5 (10- 19)	18 (9,8- 22,8)	18 (11–28)

Letter-Number Sequencing	6 (3,3- 8)	7 (5- 9)**	6,5 (3,8- 10,3)	5 (3,3- 7,8)	7 (5- 8)**	6 (4- 8,5)	5 (3,3–7)	6 (3,3- 8)	6 (5–9)

TP Dispersion Index ( %)	15 (9,7- 30,9)	12 (8,6- 16,8)	8,1 (3,8–24)	24 (13,6- 38)	21 (8,4- 39,4)	24,9 (9,3–30,8)	11,8 (5,7- 23,8)	11,5 (7- 20,3)	12 (10–13,5)
TP Work efficiency	102,5 (70,5- 119)	133,5 (103–162,5)**	156,5 (89,5–240)	76,5 (48,8- 118,5)	92 (65- 149)**	98 (75,5–131)	98 (62,8- 115,8)	119,5 (78,5–167,8)**	189 (90–204)

ROCFT- Copy	37,5 (12,5- 60)	47,5 (10- 75)	60 (17,5–92,5)	20 (10- 82,5)	12,5 (10- 38,8)	60 (10–75)	32,5 (10- 75)	52,5 (10- 75)	45 (10–75)
ROCFT- Immediate recall	10 (10- 23,8)	50 (11,3–71,9)**	33,5 (10–95)	10 (10- 15)	23,8 (10- 38,8)**	10 (10- 20)	10 (10- 25,6)	**26 (10- 58,8**)**	15 (10–75)

SF-36 Physical functioning	95 (14)	100 (5)**	90 (50)	95 (10)	100 (5)**	100 (8)	97,5 (10)	100 (5)	100 (5)
SF-36 Physical role functioning	75 (100)	100 (0)**	100 (25)	0 (94)	100 (0)**	100 (0)	12,5 (100)	100 (0)**	100 (0)
SF-36 Pain	100 (25)	100 (0)	87 (40)	100 (36)	100 (0)**	62 (48)	100 (29)	100 (0)	100 (28)
SF-36 General health perceptions	51 (29)	72 (22)**	74,5 (21)	60 (30)	77 (22)**	100 (32)	55 (14)	72 (18)**	72 (22)
SF-36 Vitality	60 (36)	90 (15)**	87,5 (24)	57,5 (33)	90 (10)**	75 (25)	70 (53)	100 (10)**	90 (10)
SF-36 Social role functioning	62,5 (50)	100 (22)**	100 (9)	56 (58)	100 (25)**	100 (13)	62,5 (60)	100 (22)**	100 (0)
SF-36 Emotional role functioning	0 (92)	100 (0)**	100 (100)	0 (92)	100 (0)**	100 (0)	0 (100)	100 (0)**	100 (0)
SF-36 Mental health	56 (46)	88 (29)**	78 (45)	44 (40)	84 (16)**	84 (26)	54 (48)	84 (16)**	84 (20)

Notes:

Within-group significant differences (Bonferroni-corrected) from Wilcoxon Signed-Rank tests are in bold and with ** (*p* < 0.025, adjusted for multiple comparisons).

Between-group significant differences (based on two-tailed Mann-Whitney U test at *p* < 0.025) are underlined.

#### Executive functions - Frontal Assessment Battery

The results of the EF assessment are described in Table 2. Within-group analysis revealed that the RC group had a better performance after intervention in FAB total score [Baseline: Mdn = 15, IQR = 14,3–16,8; Post: Mdn = 16,5, IQR = 15,3–17; (*W*_(20)_ = 105,00, *Z* = 3370, *p* < 0001, *r =* 0,75)] and its specific domains of mental flexibility [Baseline: Mdn = 2, IQR = 1–3; Post: Mdn = 3, IQR = 2–3; (*W*_(20)_ = 49,50, *Z* = 2530, *p* = 0011, *r =* 0,56)] and inhibitory control [Baseline: Mdn = 2, IQR = 2–3; Post: Mdn = 3, IQR = 2–3; (*W*_(20)_ = 77,00, *Z* = 2496, *p* = 0013, *r =* 0,56)]. The within-group analysis for the TG group also demonstrated significant improvements in FAB total score [Baseline: Mdn = 14, IQR = 12,2–16,8; Post: Mdn = 16, IQR = 14,3–17, (*W*_(20)_ = 91,00, *Z* = 3238, *p* = 0001, *r* = 0,72)] and its conceptualization [Baseline: Mdn = 2, IQR = 1,3–3; Post: Mdn = 3, IQR = 2–3; (*W*_(20)_ = 50,00, *Z* = 2496, *p* = 0013, *r =* 0,56)], and sensitivity to interference [Baseline: Mdn = 2,5, IQR = 2–3; Post: Mdn = 3, IQR = 2,3–3; (*W*_(20)_ = 40,50, *Z* = 2309, *p* = 0021, *r =* 0,52)] subtests. The TAU group also showed a statistically significant increase in FAB total score [Baseline: Mdn = 15, IQR = 13–15,8; Post: Mdn = 16, IQR = 14–17,8; (*W*_(20)_ = 120,00, *Z* = 2744, *p* = 0006, *r =* 0,61)].

#### Divided attention and processing speed - Digit Symbol Coding (DSC) and Symbol Search.

Table 2 displays the groups' scores at the different NPA moments. Results revealed that the RC group improved in both Symbol Search and DSC WAIS-III subtests: coding [Baseline: Mdn = 33, IQR = 23,5–42,3; Post: Mdn = 39, IQR = 27, 8–50; (*W*_(20)_ = 151,00, *Z* = 3530, *p* < 0001, *r* = 0,79)], incidental learning [Baseline: Mdn = 8,5, IQR = 4,5–12; Post: Mdn = 10, IQR = 6–15,5; (*W*_(20)_ = 143,00, *Z* = 3199, *p* = 0001, *r =* 0,72)] and free recall [Baseline: Mdn =6, IQR = 5–7; Post: Mdn = 7, IQR = 6,8–8; (*W*_(20)_ = 113,00, *Z* = 3068, *p* = 0002, *r* = 0,69)] and; symbol search [Baseline: Mdn = 14, IQR = 13–21,5; Post: Mdn = 19, IQR = 15–23; (*W*_(20)_ = 180,00, *Z* = 3432, *p* < 0001, *r = 0,77*)]. The TG group had significantly higher scores in free recall [Baseline: Mdn = 6, IQR = 4,3–3,7; Post: Mdn = 6,5, IQR = 6–8; (*W*_(20)_ = 93,00, *Z* = 2693, *p* = 0007, *r = 0,60*)]. The TAU group had better results in coding [Baseline: Mdn = 33,5, IQR = 24–37,8; Post: Mdn = 37, IQR = 26,3–37,8; (*W*_(20)_ = 168,00, *Z* = 2943, *p* = 0003, *r = 0,7*)].

#### Working Memory- letter-number sequencing

Concerning the working memory task, both experimental groups RC and TG revealed significant enhanced performance post intervention [RC - Baseline: Mdn = 6, IQR = 3,3- 8; Post: Mdn = 7, IQR = 5–9; (*W*_(20)_ = 24,00, *Z* = −2519, *p* = 0012, *r = 0,56*; TG - Baseline: Mdn = 5, IQR = 3,3- 7,8; Post: Mdn =7, IQR = 5- 8; (*W*_(20)_ = 25,00, *Z* = −2498, *p* = 0012, *r = 0,56*)].

#### Sustained attention- Toulouse-Piéron

Table 2 represents the results of all the groups for the sustained attention assessment. All groups improved at work efficiency index at post-intervention assessment [RC - Baseline: Mdn = 102,5, IQR = 70,5–119; Post: Mdn = 133,5, IQR = 103–162,5; (*W*_(20)_ = 206,00, *Z* = 3771, *p* < 0001, *r = 0,84*); TG - Baseline: Mdn = 76,5, IQR = 48,8–118,5; Post: Mdn = 92, IQR = 65- 149; (*W*_(20)_ = 192,00, *Z* = 3249, *p* = 0001, *r = 0,73*); TAU - Baseline: Mdn = 98, IQR = 62,8- 115,8; Post: Mdn =119,5, IQR = 78,5–167,8; (*W*_(20)_ = 169,00, *Z* = 2389, *p* = 0017, *r = 0,53*)].

#### Visual memory- Rey-Osterrieth Complex Figure Test

Table 2 illustrates the results for copy and immediate recall trials of the ROCF test for all groups. The three groups showed statistically significant superior results in the immediate recall trial at post-intervention [RC - Baseline: Mdn = 10, IQR = 10–23,8; Post: Mdn = 50, IQR = 11,3–71,9; (*W*_(20)_ = 117,00, *Z* = 3252, *p* = 0001, *r = 0,73*); TG - Baseline: Mdn = 10, IQR = 10–15; Post: Mdn = 23,8, IQR = 10–38,8; (*W*_(20)_ = 63,00, *Z* = 2678, *p* = 0007, *r = 0,59*); TAU - Baseline: Mdn = 10, IQR = 10–25,6; Post: Mdn = 26, IQR = 10–58,8; (*W*_(20)_ = 73,00, *Z* = 2682, *p* = 0007, *r = 0,59*)].

### Secondary outcome measures

#### Health, quality of life and well-being - SF-36 short form health survey

Table 2 displays the results for the eight different domains assessed by the SF-36 Health Survey. The within-groups analysis indicates that experimental group RC had significant improvements in seven of the eight domains of the SF-36 scale: physical functioning [Baseline: Mdn = 95, IQR= 86,3- 100; Post: Mdn = 100, IQR= 95–100; (*W*_(20)_ = 84,00, *Z* = 2724, *p* = 0006, *r = 0,60*)]; physical role functioning [Baseline: Mdn =75, IQR= 0- 100; Post: Mdn = 100, IQR= 100–100; (*W*_(20)_ = 53,00, *Z* = 2641, *p* = 0008, *r = 0,59*)]; general health perceptions [Baseline: Mdn = 51, IQR= 41–70; Post: Mdn = 72, IQR= 66–87; (*W*_(20)_ = 187,00, *Z* = 3709, *p* = 0001, *r =* *0,82*)]; vitality [Baseline: Mdn = 60, IQR= 42,5–78,8; Post: Mdn = 90, IQR= 85–100; (*W*_(20)_ = 171,00, *Z* = −3727, *p* = <0001, *r = 0,83)*]; social role functioning [Baseline: Mdn = 62,5, IQR= 50–100; Post: Mdn = 100, IQR= 78–100; (*W*_(20)_ = 115,50, *Z* = −3173, *p* = 0002, *r = 0,71)*]; emotional role functioning [Baseline: Mdn = 0, IQR= 0- 91,7; Post: Mdn = 100, IQR= 100- 100; (*W*_(20)_ =120,00, *Z* = −3623, *p* = <0.001, *r = 0,81*)]; and mental health [Baseline: Mdn = 56, IQR= 29–75; Post: Mdn = 88, IQR= 67–96; (*W*_(20)_ =, *Z* = −3799, *p* < 0001, *r = 0,85*)].

The TG group revealed improvements in post-intervention assessment for all of the eight domains of health and quality of life: physical functioning [Baseline: Mdn = 95, IQR= 90- 100; Post: Mdn = 100, IQR= 95–100; (*W*_(19)_ = 52,00, *Z* = 2537, *p* = 0011, *r = 0,85)*]; physical role functioning [Baseline: Mdn = 0, IQR= 0- 93,8; Post: Mdn = 100, IQR= 100–100; (*W*_(19)_ = 120,00, *Z* = 3689, *p* = 0001, *r = 0,85*)]; pain [Baseline: Mdn = 100, IQR= 65–100; Post: Mdn= 100, IQR= 100–100; (*W*_(19)_ = 28,00, *Z* = 2384, *p* = 0017, *r = 0,54*)]; general health perceptions [Baseline: Mdn = 60, IQR= 40- 70; Post: Mdn = 77, IQR= 65- 87; (*W*_(19)_ = 146,00, *Z* = 3295, *p* = 0001, *r =* *0,76*)]; vitality [Baseline: Mdn = 58, IQR= 41- 74; Post: Mdn= 90, IQR= 90- 100; (*W*_(19)_ = 171,00, *Z* = 3732, *p* = 0001, *r = 0,86*)]; social role functioning [Baseline: Mdn = 56, IQR= 38- 95; Post: Mdn= 100, IQR= 75–100; (*W*_(19)_ = 120,00, *Z* = 3428, *p* = 0001, *r = 0,79*)]; emotional role functioning [Baseline: Mdn = 0, IQR= 0- 92; Post: Mdn= 100, IQR= 100- 100; (*W*_(19)_ = 120,00, *Z* = 3571, *p* = 0001, *r = 0,81*)]; and mental health [Baseline: Mdn = 44, IQR= 26- 66; Post: Mdn= 84, IQR= 72- 88; (*W*_(19)_ = 120,00, *Z* = 3428, *p* = 0001, *r = 0,79*)].

The TAU had improvements in six of the eight subdomains of SF-36: physical role functioning [Baseline: Mdn = 12,5, IQR= 0- 100; Post: Mdn = 100, IQR= 100–100; (*W*_(20)_ = 105,00, *Z* = 3439, *p* = 0001, *r = 0,77*)]; general health perceptions [Baseline: Mdn = 55, IQR= 45- 58,8; Post: Mdn = 72, IQR= 60–77; (*W*_(20)_ = 171,00, *Z* = 3731, *p* = 0001, *r = 0,83*)]; vitality [Baseline: Mdn = 70, IQR= 36,3- 88,8; Post: Mdn = 100, IQR= 90–100; (*W*_(20)_ = 136,00, *Z* = 3521, *p* = 0001, *r = 0,78*)]; social role functioning [Baseline: Mdn = 62,5, IQR= 28- 87,5; Post: Mdn = 100, IQR= 78–100; (*W*_(20)_ = 105,00, *Z* = 3305, *p* = 0001, *r = 0,73*)]; emotional role functioning [Baseline: Mdn = 0, IQR= 0- 100; Post: Mdn = 100, IQR= 100–100; (*W*_(20)_ = 105,00, *Z* = 3490, *p* = 0001, *r = 0,78*)]; and mental health [Baseline: Mdn = 54, IQR= 34- 82; Post: Mdn = 84, IQR= 76–92; (*W*_(20)_ = 153,00, *Z* = 3626, *p* = 0001, *r = 0,81*)].

**RQ 2: Does ecologically valid cognitive training have more clinical impact in terms of cognition, health, well-being and quality of life than traditional paper-and-pencil training?**

A between-groups analysis indicated that the TG group had a more significant improvement in general cognitive functioning (MoCA`s total score) when compared with the RC group [RC: Mdn = 0,5, IQR = −1- 2; TG: Mdn = 2, IQR = 1- 4; (*U* = 113,50, *Z* = −2365, *p* = 0018, *r =* 0,37)] at post-intervention assessment.

In the processing speed assessment, namely in the Symbol Search subtest, RC had a significantly superior result, comparatively to both TG [RC: Mdn = 0,5, IQR = −1- 2; TG: Mdn = 2, IQR = 1- 4; (*U* = 113,50, *Z* = −2365, *p* = 0018, *r =* 0,37)] and TAU *(p* *= 0.019)* at six-month follow-up.

**RQ 3: Does cognitive training as a complementary intervention have an impact on abstinence maintenance?**

One of the RQs of this clinical trial was to explore the impact of the integration of cognitive training intervention, as part of the standard treatment of the alcohol abuse rehabilitation program, on the reduction of the relapse rate. To address this hypothesis, we have planned a six-month and one-year follow-up assessment for all participants involved in this study. Nonetheless, the expected difficulty in reaching this population after discharge, together with the unexpected COVID-19 pandemic contingencies, accounted for a very limited number of follow-up assessments. As such, we were only able to assess 22 participants at six months from our 60 initial sample and 11 participants at the one-year follow-up.

The within-group analyses using Wilcoxon signed tests with Bonferroni correction (α _adjusted_ =0.025), revealed no statistically significant improvements in any of the assessed cognitive measures across the groups. Nevertheless, we observed some qualitative gains in few cognitive domains six months after intervention, respectively in RC group attention subdomain (MoCA); TG group in orientation domain (MoCA) and attention (WE-TP); TAU group maintained the processing speed gains (Coding and Symbol Search) and motor programming.

A between-groups analysis revealed that the TG group maintained a superior improvement in general cognitive functioning (as assessed by MoCA) when compared to the RC group (*p* = 0.014); however, this result does not provide enough insights about the impact of CT on abstinence maintenance and should be interpreted carefully considering the reduced sample size (RC= 4; TG= 5 and TAU= 2).

## Discussion

The importance of cognitive training (CT) in the treatment of alcohol use disorder (AUD) has gained prominence in recent years (Nixon & Lewis, 2019), paralleling the growing adoption of virtual reality (VR) applications for CT purposes (Câmara et al., 2022; Faria et al., 2020; Gamito et al., 2021). This randomized controlled trial (RCT) sought to investigate the benefits of integrating a four-week CT program within the standard AUD treatment regimen. Specifically, we aimed to address the research questions that we go through in detail below.

RQ1: **Does the inclusion of cognitive training in the treatment of AUD promote the improvement of cognitive functioning, health, quality of life and well-being outcomes?**

### Primary outcome measures

Improvements in physical, emotional, and cognitive functions are frequently observed in alcohol use disorder (AUD) patients following inpatient treatment and may serve as predictors of this disorder's treatment success (Kaur et al., 2020; Lahbairi et al., 2022). Our trial findings corroborate the existing literature, as all groups exhibited statistically significant enhancements in various cognitive, health, quality of life, and well-being domains at post-intervention assessment. While abstinence alone (Bruijnen et al., 2021; Stavro et al., 2013) can partly explain some of the outcomes for all groups, it is crucial to emphasize the specific improvements achieved by both experimental groups following the CT program in comparison to the TAU group. It is important to acknowledge that spontaneous recovery of cognitive functions is not always observed in individuals with AUD. A recent meta-analysis examining the longitudinal course of cognitive functioning in this population failed to demonstrate this tendency; instead, it revealed a pervasive pattern of cognitive impairments, particularly in memory, even after long-term abstinence (Crowe et al., 2020).

In our study, the RC group presented within-group improvements at post-intervention in different cognitive domains: language (naming subtest-MoCA), executive functions (FAB total score), mental flexibility (fluency subtest-FAB), inhibitory control (Go-No-Go subtest-FAB), working memory (letter/number sequencing), processing speed (DSC coding and Symbol Search), visual memory (ROCFT immediate recall), sustained and selective attention (Work efficiency-TP). Working memory capacity has been investigated in individuals with AUD, and both experimental groups in our trial improved in this domain after intervention. Khemiri and colleagues conducted a study evaluating the impact of a five-week online WM training program on 50 participants with AUD. Like in our trial, the experimental group exhibited enhanced working memory performance compared to the control group (Khemiri et al., 2019b).

Regarding other cognitive domains typically compromised in AUD, the RC group presented improvements in domains such as mental flexibility and inhibitory control EF. Crespy and colleagues described an impaired executive profile in AUD patients evident in visuomotor processing speed, attention, and working memory tasks (Crespi et al., 2020; Khemiri et al., 2019b). Additionally, this group was the only one improving in all measures of processing speed (DS Coding and Symbol Search) and divided attention (DSC Incidental learning and free recall). As to the sustained and selective attention (TP) domain, the RC group enhanced its score after intervention in work efficiency index. The facts previously reported corroborate the ones described by Gamito and colleagues. After a VR cognitive intervention based on activities of daily living, the authors found significant improvements in attention and cognitive flexibility (Gamito et al., 2021). Concerning the improvement in the visual memory task performance, it is consistent with the results verified by Câmara and colleagues, who found the same result in visual memory post-intervention and at follow-up after a CT using the same tools used in our study with mental population (Câmara et al., 2022).

On the other hand, the paper-and-pencil group (TG) also exhibited within-group improvements in general cognitive functioning (MoCA total score), abstraction (similarities subtest FAB), executive functions (FAB total score); sensitivity to interference (conflicting instructions subtest FAB), processing speed (Symbol search), visual memory (immediate recall ROCFT and DSC free recall), working memory (letter/number sequencing), sustained and selective attention (work efficiency TP). Some of these results, such as improvement in processing speed tasks, were also found in stroke patients (Faria et al., 2020) and in a study with mental health and behavioral disorder patients, where improvements in abstraction and quality of life were reported at follow-up (Câmara et al., 2023).

Concerning the TAU group, there were enhancements in executive functions (FAB total score), abstraction (similarities FAB), processing speed (DSC Coding), sustained and selective attention (work efficiency TP), and visual memory (ROCFT-immediate recall).

We conclude that incorporating CT within a standard AUD treatment leads to greater immediate benefits (i.e., post-intervention) in cognitive domains often affected by this condition, specifically: the RC group improves in 11 (48,8 %) out of 24 assessed domains. The TG group improves in 9 (37,5 %) out of 24, whereas the TAU group improves in 5 (20,8 %) out of 24 assessed domains.

### Secondary outcome measures

In this trial, we also intended to explore the potential effects of CT benefits on quality of life and health measures (SF-36). The within-group analysis revealed that both experimental groups, RC and TG, manifested significant improvements after intervention in all questionnaire domains: physical functioning, physical role functioning, pain, general health perceptions, vitality, social role functioning, emotional role functioning, and mental health. The TAU group reported enhancements in six domains: quality of life, physical role functioning, general health perceptions, vitality, social role functioning, emotional role functioning, and mental health. The impact of AUD on the perceived health and quality of life is well documented in the literature since this kind of addictive disorder not only affects organic health and cognitive functions but also has a negative impact on all aspects of an individual's life, compromising familiar, social, labor and all functionality domains (Huang et al., 2021; Lahbairi et al., 2022; Malet et al., 2006).

RQ2: **Does ecologically valid CT reveal a more significant clinical impact on cognition, health, well-being, and quality of life compared to traditional paper-and-pencil training?**

An important goal of the technologies applied to this field of CT tools and interventions involves the development of environments that resemble the scenarios that we are used to interacting with in our daily lives. The ecological validity of the CT task and activities has been described as an important component to aggregate when developing CT systems (Faria et al., 2016, 2020).

Our second research question aimed to investigate whether the ecologically valid CT tool, Reh@City, demonstrated superior efficacy in promoting gains in cognitive, health, well-being, and quality of life outcomes when compared with a paper-and-pencil CT intervention.

The between-group analyses revealed that the TG group demonstrated a better general cognitive function (MoCA total score) performance than the RC group post-intervention. A superior performance from TG participants compared with RC participants at processing speed and abstraction domains (MoCA) was also reported by Câmara and colleagues in a study previously referred to. The authors justify this possible advantage by comparing the former CT tasks with this cognitive screening tool (Câmara et al., 2022).

Between-group differences were also verified between RC, TG and TAU regarding processing speed (Symbol Search), where RC improved more when compared to TG and TAU groups at six-month follow-up, but this result has be interpreted with caution, considering our very small sample size at follow-up assessment.

**RQ3: Does cognitive training as a complementary intervention impact abstinence maintenance?**

One of the RQs of this clinical trial was to explore the impact of the integration of cognitive training intervention, as part of the existing standard treatment of the alcohol abuse rehabilitation program, on the reduction of the relapse rate. To address this hypothesis, we have planned a six-month and one-year follow-up assessment for all participants involved in this study. Nonetheless, the expected difficulty in reaching this population after discharge and the unexpected COVID-19 pandemic contingencies accounted for a reduced number of one-year follow-up assessments. As such, we could only assess 22 participants at six months post-intervention.

A between-groups analysis revealed that the TG group maintained a superior improvement in general cognitive functioning (as assessed by MoCA) when compared to the RC group (*p* = 0.014); however, this result should be interpreted carefully considering the reduced sample size (RC= 4; TG= 5 and TAU= 2).

Regarding the long-term benefits (six-month follow-up), it is difficult to draw consistent conclusions as we had a high dropout rate at the six-month follow-up (RC = 10, TG = 5, TAU = 7). Despite the abovementioned limitation, we verify that most post-intervention gains were lost to follow-up.

Our findings align with previous studies that investigated the long-term impact of cognitive training (CT) on abstinence. While some studies have demonstrated the positive effects of CT protocols as an adjunct to traditional treatment interventions (Nixon & Lewis, 2019), other research suggests that cognitive impairments may prevail within a year of abstinence, with some cases exhibiting delayed or even absent recovery (Crowe et al., 2020; Oscar-Berman et al., 2014). In a study mentioned previously focusing on WM training in AUD, no significant differences were observed in drinking patterns overall. Nevertheless, a trend toward reduced drinking frequency among the experimental group, specifically in the number of drinks per occasion, was noted (Khemiri et al., 2019b).

We must remember the specificity of living on Madeira Island and the existing lack of supportive measures to facilitate the reintegration of AUD individuals into recovery, including social community reintegration, employment assistance, and close follow-up after treatment with family support. Additionally, other factors, such as low socioeconomic status, low literacy levels, unemployment, and limited family support, can significantly hinder abstinence. According to the existing literature (McKay, 2021), long-term abstinence is fostered by a combination of factors, including individual traits, treatment type and duration, ongoing care, social support, and engagement in activities that promote recovery (McKay, 2021).

## Limitations

Despite the positive results, several limitations of this study should be acknowledged when interpreting our findings. While the rehabilitation treatment unit Ricardo Pampuri serves a large patient population annually, our sample size for this RCT was relatively small. This limitation comes from our stringent inclusion criteria, which were designed to ensure a more homogeneous sample and control for potentially confounding variables such as the use of other substances or the presence of psychiatric disorders other than AUD. To address this limitation, future clinical trials should employ larger sample sizes, thereby enhancing statistical power and the generalizability of findings to the broader AUD patient population.

Moreover, the two psychologists who conducted the intervention sessions for both the RC and TG groups were also responsible for administering the neuropsychological assessments for all participants, raising concerns about potential unconscious bias in their evaluation of cognitive abilities. This highlights the importance of ensuring impartiality in future studies of this type by implementing a double-blind research design. While implementing a double-blind design was not feasible in the current study due to resource limitations and the impatient context, it remains a crucial consideration for future research.

Additionally, the RC and TG groups performed CT intervention using content-equivalent tools regarding personalization guidelines and difficulty progression rules. This similarity in CT approaches may account for the observed comparability of cognitive and non-cognitive outcome measures between the two groups. These equivalent results in terms of cognitive measures were not found in other similar studies.

Finally, the few follow-up results should be interpreted cautiously, as only 22 participants completed the 6-month follow-up assessment, and only 11 were re-assessed at 12 months. This high dropout rate prevents us from drawing solid conclusions regarding the long-term efficacy of the CT intervention, the sustainability of its effects, and the role of cognitive intervention as a complementary tool in AUD treatment and abstinence maintenance. Future research with higher long-term retention rates for follow-up data is crucial. Such studies would enable a more comprehensive exploration of the long-term efficacy of CT intervention in AUD treatment, to better understand cognitive fluctuation and sustained effects across different treatment time points."

## Acknowledgements

The authors want to thank Cátia Alves and Mariana Câmara for their contribution to the data collection and all the patients and health professionals from Casa de Saúde São João de Deus involved in this study.

## Funding

This work is supported by UID/04516/NOVA Laboratory for Computer Science and Informatics (NOVA LINCS) with the financial support of FCT.IP. **Additionally, it received funding from the European Union and National funds through the Institute for Supporting Small and Medium-sized Enterprises and Innovation (IAPMEI) and the Recovery and Resilience Plan under the application no 761 submitted to the measure Polos de Inovação Digital (DIH) under the terms of AAC no. 03/C16- i03/2022**.

## Declaration of competing interest

The authors declare that they have no known competing financial interests or personal relationships that could have appeared to influence the work reported in this paper.

## Appendix Supplementary materials

Image, application 1
