# Supplementary material for: Enhancing treatment outcomes of Alcohol Use Disorder patients through ICT-based cognitive training tools: a randomized controlled trial
Source: Int J Clin Health Psychol. 2025 Sep 23;25(3):100623. doi: 10.1016/j.ijchp.2025.100623 (PMC12492034; doi:10.1016/j.ijchp.2025.100623)
Supplement: Supplementary file 1 [file mmc1.docx]

| Tasks | Cognitive domain (s) | TG | Reh@City v2.0 |
| --- | --- | --- | --- |
| Cancellation | Selective and sustained attention | Identify a target stimulus (e.g., letter, number of symbol) among distractors | Identify a target item at the supermarket, pharmacy, and post-office, according to a shopping list |
| Numeric sequences | Problem-solving, numerical reasoning, and working memory | Calculate the missing numbers in a sequence considering a specific pattern (i.e., addition or subtraction) | Calculate the missing PIN numbers at the ATM |
| Problem solving | Executive functioning: Problem-solving, numerical reasoning | Solve calculations involving addition, subtraction, and multiplication, or solve arithmetic word problems presented | Select the correct invoice at the supermarket |
| Association | Visual memory, conceptualization | Pair associated images | Find matching cards in a memory game at the park |
| Comprehension of contexts | Comprehension, reasoning | Analyze a target image and find the correct descriptions by answering true or false affirmations | Not applicable |
| Image pairs | Visual memory | Memorize a set of image pairs and then recall them after a 30-minute interval | Find matching cards in a memory game at the park |
| Word search | Selective attention | Find target words that can be placed horizontally, vertically, or diagonally among random letters | Not applicable |
| Mazes | Executive functioning- Planning, spatial orientation, and problem-solving | Find the route from the start to the exit of a labyrinth | Find the shortest navigation route to arrive to a given location in the virtual city |
| Categorization | Executive functioning- categorization and conceptualization | Identify the category of different images | Select target items from a clothing store according to a given category (e.g., shoes, sunglasses, women’s clothing) |
| Action sequencing | Executive functioning- planning, sequencing | Organize a set of actions to perform a given activity | Select, in the proper order, the steps needed to accomplish an activity of daily living at home |
| Memory of stories or pictures | Verbal memory, visual memory, comprehension, and working memory | Memorize information about a story or picture and recall it after a few minutes by answering true or false questions | Memorize verbal or visual information from a newspaper or magazine at the kiosk and then answer true or false questions, regarding the previous information, when reaching the next location |

Supplementary material A: Paper-and-pencil tasks (TG) correspondence to VR tasks (Reh@city v2.0) and respective cognitive domains- table adapted from (Faria, Pinho & Bermúdez i Badia, 2020; Câmara et al., 2021).
